# Multiple approaches to microbial source tracking in tropical northern Australia

**DOI:** 10.1002/mbo3.209

**Published:** 2014-09-16

**Authors:** Matthew Neave, Heidi Luter, Anna Padovan, Simon Townsend, Xavier Schobben, Karen Gibb

**Affiliations:** 1 Research Institute for the Environment and Livelihoods, Charles Darwin UniversityCasuarina, Northern Territory, Australia; 2 Northern Australian Marine Research Alliance, Arafura Timor Research Facility DarwinBrinkin, Northern Territory, Australia; 3 Department of Land Resource Management, Northern Territory GovernmentPalmerston, Northern Territory, Australia; 4 Department of Health, Northern Territory GovernmentCasuarina, Northern Territory, Australia

**Keywords:** 454 Pyrosequencing, Australia, bacteria, DGGE, fecal, sewage, tracking, tropical

## Abstract

Microbial source tracking is an area of research in which multiple approaches are used to identify the sources of elevated bacterial concentrations in recreational lakes and beaches. At our study location in Darwin, northern Australia, water quality in the harbor is generally good, however dry-season beach closures due to elevated *Escherichia coli* and enterococci counts are a cause for concern. The sources of these high bacteria counts are currently unknown. To address this, we sampled sewage outfalls, other potential inputs, such as urban rivers and drains, and surrounding beaches, and used genetic fingerprints from *E. coli* and enterococci communities, fecal markers and 454 pyrosequencing to track contamination sources. A sewage effluent outfall (Larrakeyah discharge) was a source of bacteria, including fecal bacteria that impacted nearby beaches. Two other treated effluent discharges did not appear to influence sites other than those directly adjacent. Several beaches contained fecal indicator bacteria that likely originated from urban rivers and creeks within the catchment. Generally, connectivity between the sites was observed within distinct geographical locations and it appeared that most of the bacterial contamination on Darwin beaches was confined to local sources.

## Introduction

Water quality testing of recreational beaches has traditionally been based on counts of *Escherichia coli* (*E. coli*) or enterococci, and their presence was taken to indicate sewage contamination (Harwood et al. 2013). A fundamental problem with these conventional water quality tests is that the contamination can originate from a variety of sources (Layton et al. 2009; McLellan et al. 2013). As a consequence, even if the test is “positive” for *E. coli* or enterococci, we do not know whether the contamination is environmental or human fecal derived. This is important because water contaminated with human-derived effluent generally contain more human-specific pathogens (Scott et al. 2002; Soller et al. 2010), and therefore, may pose a greater risk to human health.

There are several reasons why the conventional indicators are not always reliable indicators of human fecal contamination: many warm- and cold-blooded animals contain indicator bacteria in their feces (Rana et al. 2011); indicator bacteria are not well correlated with human pathogens or pathogen survival profiles (Santiago-Rodríguez et al. 2010); the historically popular indicators can grow naturally in the environment in habitats such as ponds, beach sand, soil and plant cavities (Layton et al. 2009; Whitman et al. 2009; Santiago-Rodríguez et al. 2010); and there is evidence that these strains have evolved as unique environmental strains (Dobrindt et al. 2004). The real challenge is whether the mix of strains that are counted as *E. coli* or enterococci in conventional tests, and which are also identified in biomarker assays, have a genetic fingerprint that can be traced to the source.

To reduce the genetic “noise” and to increase confidence in source identification, multiple lines of evidence are needed, ranging from *E. coli* and enterococci, which are not always reliable for source tracking, through to markers developed in recent molecular biology research. The latter are not well known in conventional testing regimes because they are anaerobes that do not grow in plate culture. Many of the recently proposed indicator bacteria have been identified in the Human Microbiome Project as the dominant bacteria in feces (e. g., Arumugam et al., 2011) and typically belong to the *Bacteroidales, Bifidobacterium, Clostridiaceae, Lachnospiraceae* or *Ruminococcaceae* (Mieszkin et al. 2009; Silkie and Nelson 2009; McLellan et al. 2010, 2013; Newton et al. 2011, 2013; McQuaig et al. 2012; Harwood et al. 2013). The use of multiple species and techniques to obtain several lines of evidence is now considered an important component of effective microbial source tracking (Harwood et al. 2013).

There are few microbial source-tracking studies of macro-tidal harbors in the wet-dry tropics (but see Toledo-Hernandez et al. 2013). Differences in temperature, rainfall, salinity and solar radiation in the tropics are likely to have an important influence on the survival profiles of fecal bacteria compared to more temperate environments. At our tropical study location in Darwin, northern Australia, the harbor generally has good water quality except for a few locations that periodically have high bacterial counts (AHU 2012). In 2010 and 2011, local beaches were closed on multiple occasions in the dry season due to elevated counts of *E. coli* and enterococci. Although there were concerns about sewage discharges and other suspected inputs, such as urban rivers and drains, the source was unknown. The contamination may have originated from a point source, such as a waste treatment plant, or a diffuse, intermittent and indirect route, that is, contamination from surrounding urban areas and agricultural land that may include feces from humans and other animals. Furthermore, environmental strains may have contributed to elevated counts.

To address these unknowns, thirty sites in the Darwin region were sampled at the expected peak of dry season fecal indicator counts (based on previous surveys). The sites included three sewage outfalls, other potential inputs (such as urban rivers and drains), beaches that had previously recorded high bacterial counts and beaches previously unaffected. Similarities between the *E. coli* and enterococci communities were measured using denaturing gradient gel electrophoresis (DGGE) and 454 pyrosequencing was used to examine the total bacterial community. Specific fecal markers were detected by polymerase chain reaction (PCR) and used to identify contamination that was likely to be human. We used the *Enterococcus faecium esp* fecal marker, although early reports that it was human specific (Scott et al. 2005), were later disproved when it was amplified from dogs and captive seals (Layton et al. 2009). *Aeromonas* was also selected as a fecal biomarker after Janda (1991) and Janda and Abbott (1998) reported that fecal isolates from humans with gastrointestinal disease predominantly contained *A. hydrophila*, *A. caviae*, and *A. veronii*, which confirmed their status as enteropathogens. The *Bacteroides thetaiotaomicron* fecal marker was included because it is considered to be mostly human specific (Teng et al. 2004; Aslan and Rose 2013). In addition, the 454 pyrosequencing data was explored for potentially useful indicator bacteria for the Darwin population. We predicted that through using this multifaceted approach we would be able to track sources of contamination on Darwin beaches and uncover new markers for this area and other tropical regions.

## Experimental Procedures

### Sites

**Figure 1:**
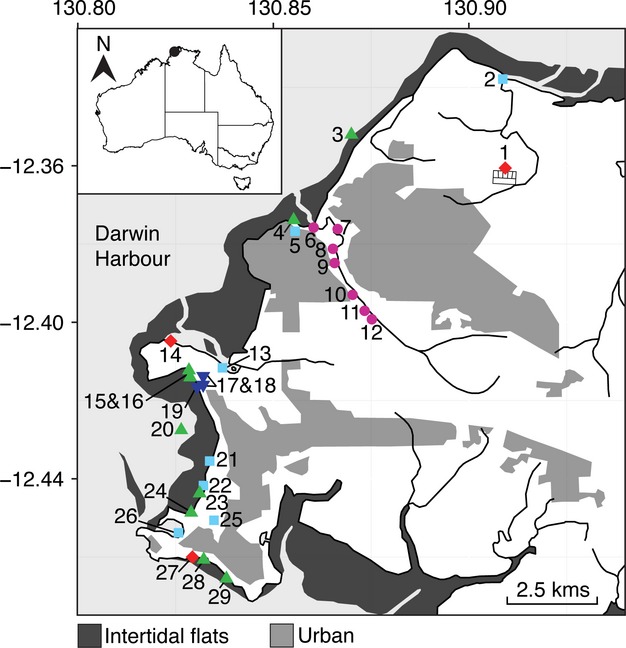
Site locations in the Darwin catchment, northern Australia. Red diamonds are the sewage discharge sites, light blue squares are other suspected inputs, green triangles are the beaches, pink circles are Rapid Creek and dark blue inverted triangles are Lake Alexander. For site names and co-ordinates see Table 1. The control site 30 “Wagait Beach” west of Darwin Harbour is not shown. This figure was created using ggplot2 (Wickham 2009) in R (R Core Team 2013) using data from Geoscience Australia (2006).

Thirty in-shore sites were selected in Darwin Harbor (Fig. 1; Table 1) and included beaches subject to high bacterial concentrations at sites 4, 15, 16, 23, 24 and 29. Two beaches at sites 3 and 30 were considered reference beaches that had never previously had elevated bacterial counts. Three sewage outfalls (sites 1, 14, and 27) were included, each with different sewage treatment strategies. The Leanyer-Sanderson outfall (site 1) is treated using a pond (secondary) treatment process with surface aeration, the Ludmilla outfall (site 14) by an enhanced primary treatment consisting of screening, grit removal and precipitation of suspended and coagulated solids using chemicals (including chlorine), and Larrakeyah outfall (site 27) by maceration only. Other suspected inputs, such as urban rivers and drains, were included because of previous high bacteria counts, or because of their proximity to outfalls, to hobby farms or areas with high fertilizer use. There were multiple sites along the suburban “Rapid Creek” because there was a view it was involved in the closure of Rapid Creek Beach (site 4). Other sites included storm water drains at Chapman Rd (site 5), Botanic Garden (site 22), a golf pond (site 25) and a marine lake (Lake Alexander sites 17 and 18).

**Table 1:** Site information and average *Escherichia coli,* enterococci and fecal coliform counts

Site no.	Location	Latitude	Longitude	Category	*E. coli* (CFU/100 mL)	Enterococci (CFU/100 mL)	Fecal coliforms (CFU/100 mL)
**1**	**Leanyer-Sanderson outfall**	**−12.3606075**	**130.9092132**	**Sewage outfall**	**1800**	**59**	**2550**
2	Buffalo Ck mouth	−12.3379706	130.9085343	Suspected source	10	22	11
3	Casuarina Beach	−12.3521035	130.8699403	Beach	0	11	0
4	Rapid Ck Beach	−12.3742735	130.8557267	Beach	6	51	7
5	Stormwater drain to Rapid Ck	−12.3764050	130.8559717	Suspected source	170	209	1260
6	Rapid Ck 6 Mouth	−12.3759401	130.8605198	Rapid Creek	115	143	195
7	CDU channel into Rapid Creek	−12.3762815	130.8660942	Rapid Creek	175	440	205
8	Rapid Ck 4	−12.3808526	130.8651428	Rapid Creek	550	370	10,800
9	Rapid Ck 5 ds Tide Gauge	−12.3847275	130.8655381	Rapid Creek	1000	1570	100,000
10	Rapid Ck 5 ds McMillans Road	−12.3929836	130.8702496	Rapid Creek	280	380	280
11	Rapid Ck 1 near Airport	−12.3992425	130.8751722	Rapid Creek	3	37	3
12	Rapid Ck 4 Yankee Pool	−12.3971267	130.8733333	Rapid Creek	5	32	5
13	Ludmilla Ck mouth	−12.4121370	130.8371519	Suspected source	165	66	165
14	**Ludmilla outfall**	**−12.4055194**	**130.8239278**	**Sewage outfall**	**2**	**0**	**2**
15	Fannie Bay bch	−12.4123575	130.8286103	Beach	0	6	0
16	Fannie Bay bch	−12.4128964	130.8291199	Beach	7	40	7
17	Lake Alexander NE	−12.4137374	130.8317282	Lake Alexander	0	1	0
18	Lake Alexander SW	−12.4161860	130.8318741	Lake Alexander	1	14	1
19	Lake Alexander intake	−12.4170756	130.8313194	Lake Alexander	125	11	140
20	Boat Clubs	−12.4271063	130.8263314	Beach	0	0	0
21	Vesteys Ck	−12.4351733	130.8337085	Suspected source	127	530	127
22	Botanic Garden Drain	−12.4418887	130.8324496	Suspected source	845	630	845
23	Mindil Beach	−12.4433762	130.8316967	Beach	18	270	23
24	Little Mindil	−12.4488712	130.8291140	Beach	13	11	14
25	Golf Pond	−12.4502050	130.8350373	Suspected source	95	45	95
26	Cullen Bay	−12.4538669	130.8255159	Suspected source	0	17	0
**27**	**Larrakeyah outfall**	**−12.4600417**	**130.8293394**	**Sewage outfall**	**9,000,000**	**915,000**	**10,000,000**
28	Doctors Gully	−12.4607458	130.8321497	Beach	39	14	99
29	Lameroo Beach	−12.4657582	130.8380808	Beach	29	14	130
30	Wagait Beach	−12.4265594	130.7363273	Beach	0	0	0

Sewage outfalls are bolded.

### Field collection and sample handling

**Figure 2:**
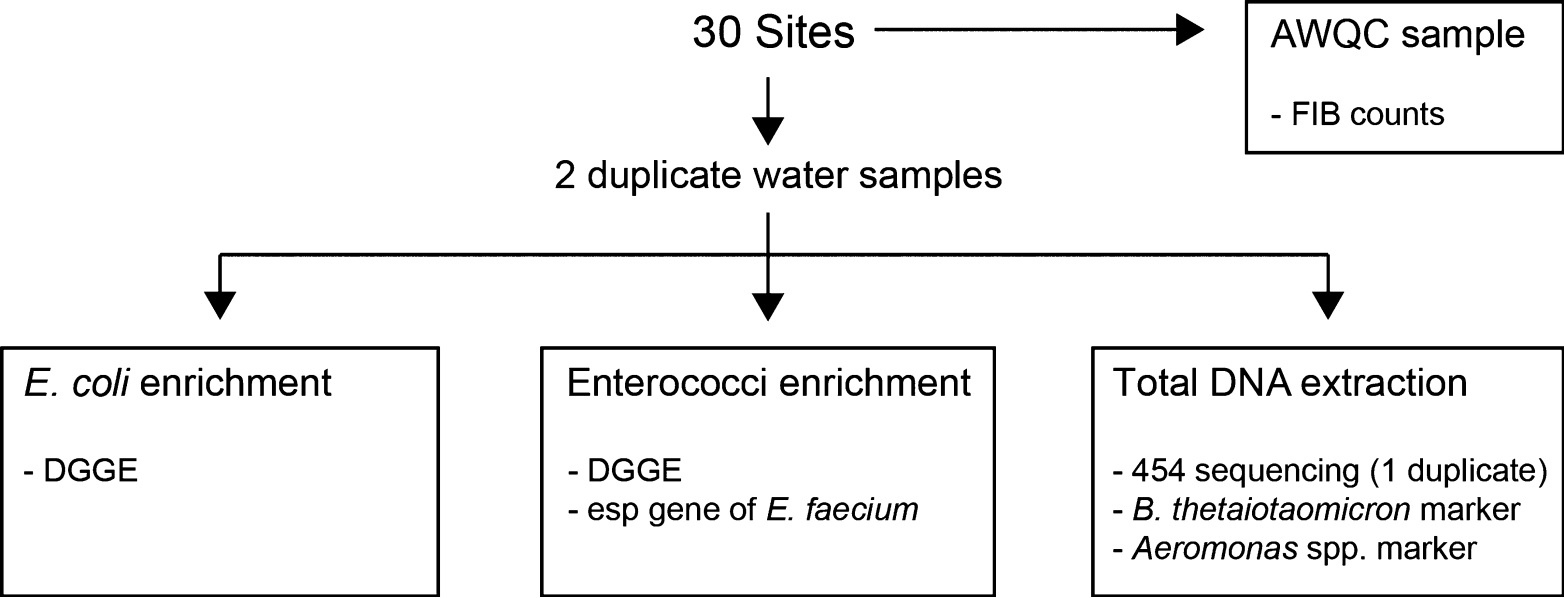
Schematic of the sampling and experimental procedures.

Water samples for bacterial community analysis were collected in duplicate from 30 sites in Darwin Harbor (Fig. 1; Table 1). A schematic of the processing procedures is given in Figure 2. The sites were sampled at approximately the same time (3 h after a spring high tide) on June 20, 2011. At the outfalls samples were taken before mixing with the receiving waters. The samples were obtained by inverting a sterile, 1 L bottle ˜20 cm below the water surface. The samples were then placed on ice and taken to the laboratory for analysis. An additional 250 mL of water was collected at each site, kept on ice, and sent to the Australian Water Quality Centre (AWQC) in Adelaide, South Australia. At the AWQC, samples were tested for *E. coli*, enterococci and fecal coliforms using membrane filtration in accordance with Australian/New Zealand Standards (AS/NZS 4276.5). Briefly, 100 mL of water was filtered through a 0.45 *μ*m membrane filter, which was then placed on membrane lauryl sulfate media at 36 ± 2°C. Colonies were enumerated and results were expressed as colony-forming units per 100 mL (CFU/100 mL). At each site, turbidity, total suspended solids (TSS), temperature, dissolved oxygen (DO) and electrical conductivity (EC) were measured using a Hydrolab Datasonde 4a (Austin, TX, USA) and a YSI 6-Series sonde (Yellow Springs, OH, USA). Total nitrogen (TN), total phosphorous (TP), nitrite (NO_2_), nitrate (NO_3_), and ammonia (NH_3_) were measured in filtered water samples using flow injection analysis (FIA) according to standard methods (APHA 1989).

### Total bacterial DNA, and *E. coli* and enterococci enriched DNA

The water samples for bacterial community analysis were processed within 6 hours of sample collection. Each of the 1 L water samples was divided into 3 × 300 mL portions: two were used for the enrichment of *E. coli* and enterococci and one was used for total DNA extraction (Fig. 2). The 300 mL portions were filtered through sterile, nitrocellulose membranes (0.45 *μ*m pore size) before either enrichment or total DNA extraction. For *E. coli* enrichment, the membranes were transferred to modified m-TEC agar plates (Difco, Sparks, MD, USA) and incubated for 16 h at 44.5°C (Esseili et al. 2008). For enterococci enrichment, the membranes were transferred to membrane-Enterococcus indoxyl-*β*-d-glucoside (mEI) agar (Difco, Sparks, MD, USA) and incubated for 24 h at 41°C. Following the incubations, the number of colonies was recorded using an index of colony abundance. The filters were then removed from the culture medium and the DNA was extracted using the PowerWater DNA Isolation Kit (MoBio, Carlsbad, CA, USA) according to the manufacturer's instructions. The third, unenriched filter was transferred directly to the PowerWater DNA Isolation Kit (MoBio, Carlsbad, CA, USA) and the total DNA was extracted according to the manufacturer's instructions.

### *E. coli* and enterococci community signatures

Three genes that are useful in differentiating *E. coli* communities (Esseili et al. 2008) were used to examine *E. coli* community diversity: malate dehydrogenase (*mdh*), *β*-3-D-glucuronidase (*uidA*), and an outer membrane phosphoporin (*phoE)*. These genes were amplified (Table S1) from the *E. coli* enriched samples and separated using DGGE. PCR products were generated in triplicate, 50 *μ*L reactions (Sahara PCR mix; Bioline, Taunton, MA, USA) to ensure that a representative bacterial sample was obtained. Optimal DGGE conditions for the *mdh* and *uidA* genes was a denaturant gradient of 50–70% and for the *phoE* gene, the best pattern was obtained with a gradient of 20–35%. PCR amplicons were separated at 75 volts for 17 h at 60°C using the phorU System (Ingeny, Goes, The Netherlands). The separated DNA fragments were then stained using SYBR Gold Nucleic Acid Gel Stain (Invitrogen, Carlsbad, CA, USA) and visualized under UV light. For the enterococci enriched samples, a DGGE marker was developed based on the elongation factor EF-Tu (*tuf*) gene because it can detect enterococci at the genus level (Ke et al. 1999) and has been used for DGGE with other bacteria (Kassem et al. 2011). Primers and PCR conditions were optimized (Table S1) and the best DGGE denaturant range was 40–60%. Electrophoresis separations and DNA visualization were as described above.

### Fecal markers

The enterococci-enriched DNA samples were tested for the *E. faecium esp* faecal marker (Table S1). Total DNA extracted from the water samples were tested for *Aeromonas* spp. using the *Aeromonas* cytolytic aerolysin (*Aero*) gene and for *B. thetaiotaomicron* using 16S rRNA primers (Table S1). Selected amplicons from the samples were sequenced to check their identity. Sequencing reactions were compiled using the Big Dye Terminator Kit, version 3.1 Applied Biosystems, Foster City, CA, USA. The reactions contained 4 *μ*L of either forward or reverse primer (0.8 pmol/*μ*L), 1 *μ*L of big dye terminator enzyme, 3.5 *μ*L of 5x sequencing buffer and 5–10 ng of template DNA in a 20 *μ*L reaction. The sequencing reactions were cycled through 94°C for 300 sec, followed by 30 cycles of 96°C for 10 sec, 50°C for 5 sec and 64°C for 240 sec. Products were then precipitated and sequenced in both directions using a Genetic Analyzer 3130XL Applied Biosystems, Foster City, CA, USA. The consensus sequence was obtained, using MacVector, version 10.5 (MacVector, Inc. Cary, NC, USA).

### 454 16S rRNA pyrosequencing

The bacterial 16S rRNA hypervariable V4-region was amplified by PCR from one total DNA water sample at each of the 30 sites using the A-563F (Claesson et al. 2010) and B-1046R primers (Sogin et al. 2006). Primer sequences contained barcode sequences (Parameswaran et al. 2007) and a Roche 454 adaptor sequence (Roche Diagnostics, Castle Hill, Australia). Using the Roche FastStart High Fidelity PCR System, each PCR reaction (four separate reactions per sample) comprised 1 *μ*L template DNA, 1.8 mmol/L MgCl_2_, 0.2 mmol/L dNTPs, 0.8 *μ*mol/L primers, 0.5 *μ*L of FastStart High Fidelity Enzyme Blend in a total volume of 50 *μ*L. PCR conditions were 92°C for 2 min and 30 cycles of 94°C for 30 sec, 57°C for 45 sec, 72°C for 1 min, with a final extension of 72°C for 10 min. The four replicate PCR reactions for each sample were pooled, and then purified using the QIAquick PCR Purification Kit (Qiagen, Chadstone, Australia). PCR products were quantified on a 2% agarose gel and sequenced using a Roche GS FLX (454) sequencer at the Australian Genome Research Facility (AGRF) in Brisbane, QLD, Australia.

Pyrosequencing flowgram files (SFF) from AGRF were processed using Mothur (Schloss et al. 2009). Flowgrams were filtered and denoised using the AmpliconNoise (Quince et al., 2011) function within Mothur. If sequences were <200 bp, contained ambiguous characters, had homopolymers longer than 8 bp, more than one MID mismatch, or more than two mismatches to the reverse primer sequence, they were removed from the analysis. Sequences deemed unique by Mothur were aligned against a SILVA alignment (http://www.mothur.org/wiki/Silva_reference_alignment). Chimeric sequences were removed using UCHIME (Edgar et al. 2011) and grouped into 97% operational taxonomic units (OTUs) based on pairwise distance matrices created in Mothur. OTUs were classified in Mothur using the SILVA database (Quast et al. 2013). Venn diagrams were created in Mothur using the *venn* command. The normalized shared file was used for statistical analyses.

### Network analyses

The Mothur shared file was converted to a Cytoscape network file using a custom R script (available on request). The dataset was trimmed to the top 5000 most abundant OTUs and singleton reads were removed to reduce complexity. The network was constructed as a bipartite graph, containing both OTUs and sites as nodes, and edges were drawn between OTUs and the site in which they were detected. The weight of the edge was proportional to the abundance of the OTU. The networks were visualized using Cytoscape v2.8.3 (Smoot et al. 2011). The edge-weight spring-embedded algorithm as implemented in Cytoscape was used to cluster the nodes, where nodes repel each other and edge connections act as springs pulling nodes together.

### SourceTracker

We used SourceTracker v0.9.5 (Knights et al. 2011) as a Bayesian approach to estimating proportions of OTUs from the suspected inputs that were detected on the beaches. The complete Mothur shared file from above was used for this analysis. All beaches were designated as sinks and all other sites as sources. SourceTracker was run with the default settings and an alpha of 0.001.

### Statistical analyses

DGGE fingerprint patterns were photographed and then analyzed using GelCompar II software (version 6.5; Applied Maths NV, Sint-Martens-Latem, Belgium). A similarity matrix of the patterns was obtained using 1% optimization and 1% position tolerance and the Dice band-based coincidence index. Cluster analysis was then performed using the unweighted pair group method with arithmetic means (UPGMA) algorithm. The results were displayed using dendograms to visually show similarities among samples.

## Results

### *E. coli* and enterococci concentrations

**Figure 3:**
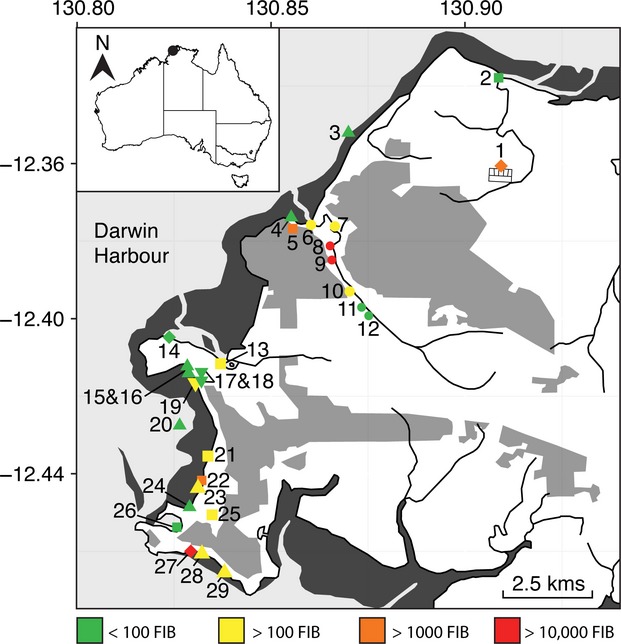
Sites overlaid with an indication of faecal indicator bacteria (FIB) counts per 100 mL of water. FIB counts were obtained by the addition of *E. coli*, enterococci, and fecal coliform counts in Table 1. Diamonds are the sewage discharge sites, squares are other inputs, triangles are the beaches, circles are Rapid Creek and inverted triangles are Lake Alexander. The control site 30 “Wagait Beach” west of Darwin Harbour is not shown and had a FIB count of <100.

*E. coli,* enterococci and fecal coliform concentrations for each of the 30 study sites (Fig. 1) are detailed in Table 1 and illustrated in Figure 3. Elevated bacterial counts were detected at the Leanyer-Sanderson sewage outfall (site 1) but not in its receiving waters (site 2). The Larrakeyah sewage outfall (site 27) had very high counts, while the bacterial counts at nearby Doctors Gully (site 28) and Lameroo Beach (site 29) were slightly elevated. The third sewage outfall (Ludmilla; site 14) had very low counts, probably due to the chlorine gas treatment used at the plant. A cluster of high readings occurred in the lower reaches of Rapid Creek (sites 6–10), although counts at the adjacent Rapid Creek Beach (site 4) were low. Another cluster of higher readings was seen for Mindil Beach (site 23) and several drains and waterways that flow onto the beach (sites 21, 22, and 25). Fannie Bay beach (sites 15 and 16) had high bacterial counts in the past, however, on our day of sampling counts were low. The two beaches selected as references (sites 3 and 30) had very low bacterial counts.

### Water quality and nutrients

The three sewage outfalls had higher turbidity, higher TSS and lower DO than the other sites (Table S2). Of the three sewage outfalls, site 14 (Ludmilla) and site 27 (Larrakeyah), had similar water quality and nutrient profiles compared to site 1 (Leanyer Sanderson). Rapid Creek sites (10, 11, and 12) upstream from a weir were different from the other sites by lower salinity and pH, and higher turbidity. The Rapid Creek sites below the weir (sites 6–9) had similar physical data to the beaches and the suspected sources. The remaining sites had relatively similar physical environmental data to each other.

Nutrients at the three outfalls (1, 14, and 27) were higher than at the other sites (Table S3). However, there were differences in nutrient loadings within the treatment plants. Ludmilla (site 14) and Larrakeyah (site 27) had similar nutrient profiles to each other, with higher levels of TN, TP, and ammonia, and lower levels of nitrate and nitrite compared to Leanyer-Sanderson (site 1), which had much higher concentrations of nitrate and nitrite and lower levels of the other nutrients. The remaining sites showed little difference in their nutrient profiles.

### Tracking contamination using *E. coli* community signatures

Water samples enriched for *E. coli* were analyzed using three *E. coli* markers: *uid-A, mdh* and *phoE* (Figs. S1–S3). These genes have previously been useful for differentiating *E. coli* from different hosts (Esseili et al. 2008). In this case, however, all three markers produced complex DGGE patterns and no clear associations between samples emerged. For example, using the *uid-A* gene, the Ludmilla and Larrakeyah outfalls (sites 14 and 27) grouped together and to one Doctor's Gully sample (site 28), however, the duplicate for site 28 was different from these three.

### Tracking contamination using enterococci community signatures

**Figure 4:**
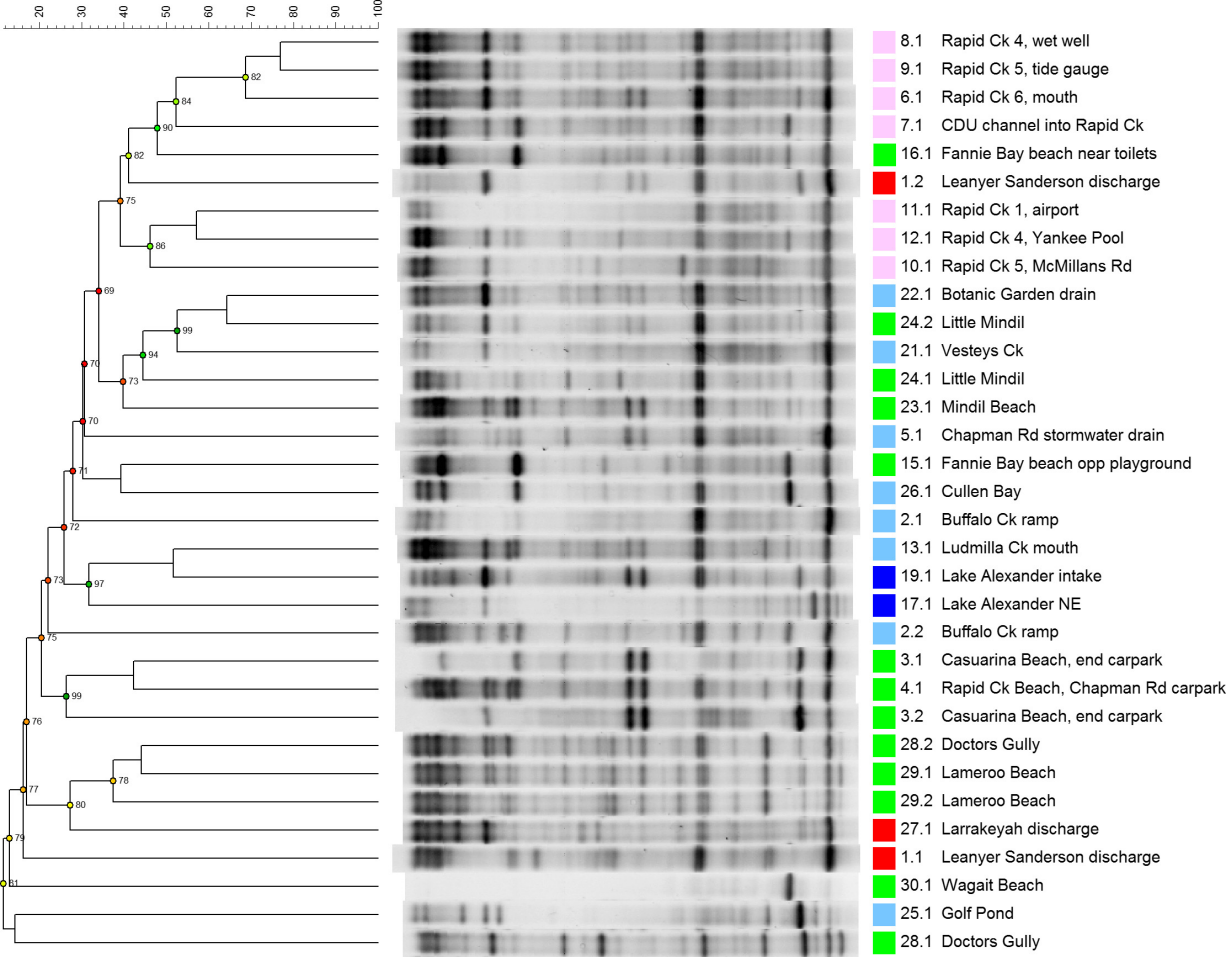
DGGE separation of the *tuf* gene in enterococci enriched samples. Duplicate samples are only shown if they are different. Beaches are green, Lake Alexander is dark blue, other inputs are light blue, Rapid Creek is pink and the discharges are red. The branch numbers signify the cophenetic correlation value.

The *tuf* gene was amplified from samples enriched for enterococci and separated using DGGE (Fig. 4). The signature for enterococci was less complex than for *E. coli* and reasonably informative. The Larrakeyah sewage outfall (site 27) had a similar enterococci community profile to nearby beaches at Doctors Gully (site 28) and Lameroo Beach (site 29), while the enterococci community from the Leanyer-Sanderson sewage outfall (site 1) did not match nearby beaches (site 2) and replicate samples from this outfall were variable. The third sewage outfall (Ludmilla; site 14) had no profile because no colonies grew on the enterococci-specific media plates, probably due to the chlorine gas treatment at this plant. Sites in the lower reaches of Rapid Creek (sites 6–9) had a similar profile to each other, and Mindil Beach (sites 23 and 24) were similar to several nearby creeks and drains (sites 21 and 22). The distinct geographical groupings of the enterococci community profiles, i.e., Larrakeyah (sites 27–29), Mindil Beach (sites 21–24) and lower Rapid Creek (sites 6–9), resemble the clusters of high bacterial counts in Table 1 and Figure 3.

### Detecting contamination using fecal markers

Water samples were tested using three fecal markers (Table 2). *Bacteroides thetaiotamicron* and *E. faecium* were tested for host specificity using DNA extracted from the feces of 24 native, introduced and domestic animal species. *B. thetaiotamicron* was negative for all non-human samples except for one species of frog and *E. faecium* was negative for all non-human samples except for one species of wallaby, one species of wallaroo and a monkey. The Larrakeyah sewage outfall (site 27) was positive for all markers and all replicates and adjacent beaches were also frequently positive (sites 28 and 29). The Leanyer-Sanderson outfall (site 1) was only positive for *B. thetaiotamicron* and it was not detected in receiving waters (site 2). The Ludmilla sewage outfall (site 14) was positive for two of the markers, which were also detected in Ludmilla Creek (site 13). Lower Rapid Creek (sites 6–9) was positive for *E. faecium* esp and *Aeromonas*, and occasionally positive for *B. thetaiotamicron*. The beach near the estuary of Rapid Creek (site 4) was also positive for *E. faecium* esp and *Aeromonas*. The waterways leading to Mindil Beach, i.e. Vesteys Creek (site 21) and the Botanic gardens drain (site 22), were positive for the fecal markers, although Mindil Beach (sites 22 and 23) were positive for only one (different) marker at each site. The clusters of positive results surrounding the Larrakeyah discharge, lower Rapid Creek and Mindil Beach reflect the results in Table 1, and Figures 3 and 4.

**Table 2:** Detection of fecal bacteria in the water samples

Site	*Enterococcus faecium*	*Aeromonas* spp.	*Bacteroides thetaiotaomicron*
**1. Leanyer-Sanderson discharge**	− −	− −	**+ +**
2. Buffalo Creek ramp	− −	+ +	− −
3. Casuarina Beach	− −	+ +	− −
4. Rapid Creek Beach	− +	+ +	− −
5. Chapman Rd stormwater drain	+ +	− −	− −
6. Rapid Creek 6, mouth	+ +	+ +	− −
7. CDU channel	+ +	+ +	− −
8. Rapid Creek 4, wet well	+ +	+ +	+ −
9. Rapid Creek 5, tide gauge	+ +	+ +	− −
10. Rapid Creek 5, McMillans Rd	− −	− −	+ −
11. Rapid Creek 1, near airport	− −	− −	− −
12. Rapid Creek 4, Yankee Pool	− −	− −	− −
13. Ludmilla Creek	+ +	− −	+ +
**14. Ludmilla discharge**	− −	**+ +**	**+ +**
15. Fannie Bay Beach, playground	− −	− −	− −
16. Fannie Bay Beach, toilets	− −	− −	+ −
17. Lake Alexander NE	− −	− −	− −
18. Lake Alexander SW	− −	− −	− −
19. Lake Alexander intake	− −	− −	− −
20. Boat clubs	− −	− −	+ −
21. Vesteys Creek	− −	+ −	+ +
22. Botanic Garden drain	+ +	+ +	+ −
23. Mindil Beach	− −	+ −	− −
24. Little Mindil	− −	− −	+ −
25. Golf Pond	− −	− −	− −
26. Cullen Bay	− −	+ +	+ −
**27. Larrakeyah discharge**	**+ +**	**+ +**	**+ +**
28. Doctors Gully	+ −	+ +	+ −
29. Lameroo Beach	− −	+ +	+ −
30. Wagait Beach	− −	− −	− −

+ −, one duplicate was positive; + +, both duplicates were positive; − −, both duplicates were negative. Sewage outfalls are bolded.

PCR amplicons for each fecal marker were sequenced and matched against sequences in the Genbank sequence database (http://www.ncbi.nlm.nih.gov/genbank). Two types of *E. faecium* esp were detected in the Darwin samples: a rare type that was only detected in the Botanic Garden drain (both duplicates) and an abundant type that was detected in all other positive samples (GenBank #KF955968-KF955982). The *Aeromonas* spp. amplicon matched the pathogen *A. hydrophila*, however more than one strain of the pathogen matched our sequences, and some of our sequences were slightly different from each other (GenBank #KF955963-KF955967). Samples that were positive for *B. thetaiotaomicron* (GenBank #KF955983-KF955986) were a match to the *B. thetaiotaomicron* isolate in Genbank.

### 454 pyrosequencing for microbial source tracking

Following processing of the 454 pyrosequencing data set, there were 264,832 reads from the 30 samples. The rarefaction curves for the beaches, sewage outfalls and Lake Alexander appeared to be reaching a plateau but this was not the case for Rapid Creek and the other inputs (Fig. S4).

**Figure 5:**
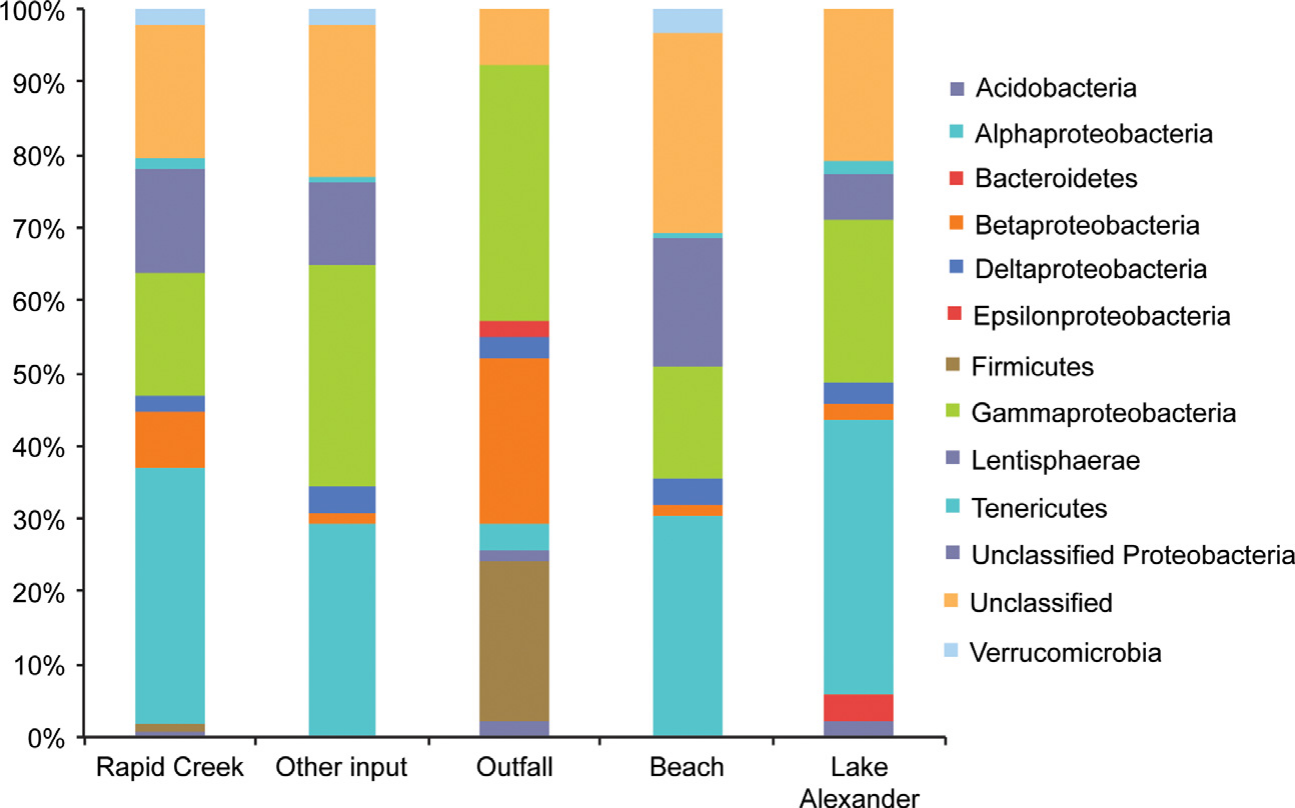
The 50 top ranked OTUs (at 97% similarity) in water for each sample category. Classification was to phyla except Proteobacteria, which were resolved to class.

The microbial community at the phylum level was similar for all site types, except for the outfalls (Fig. 5). The outfalls were different due to higher proportions of Firmicutes and Betaproteobacteria, while the other sites were dominated by Alphaproteobacteria and Gammaproteobacteria.

**Figure 6:**
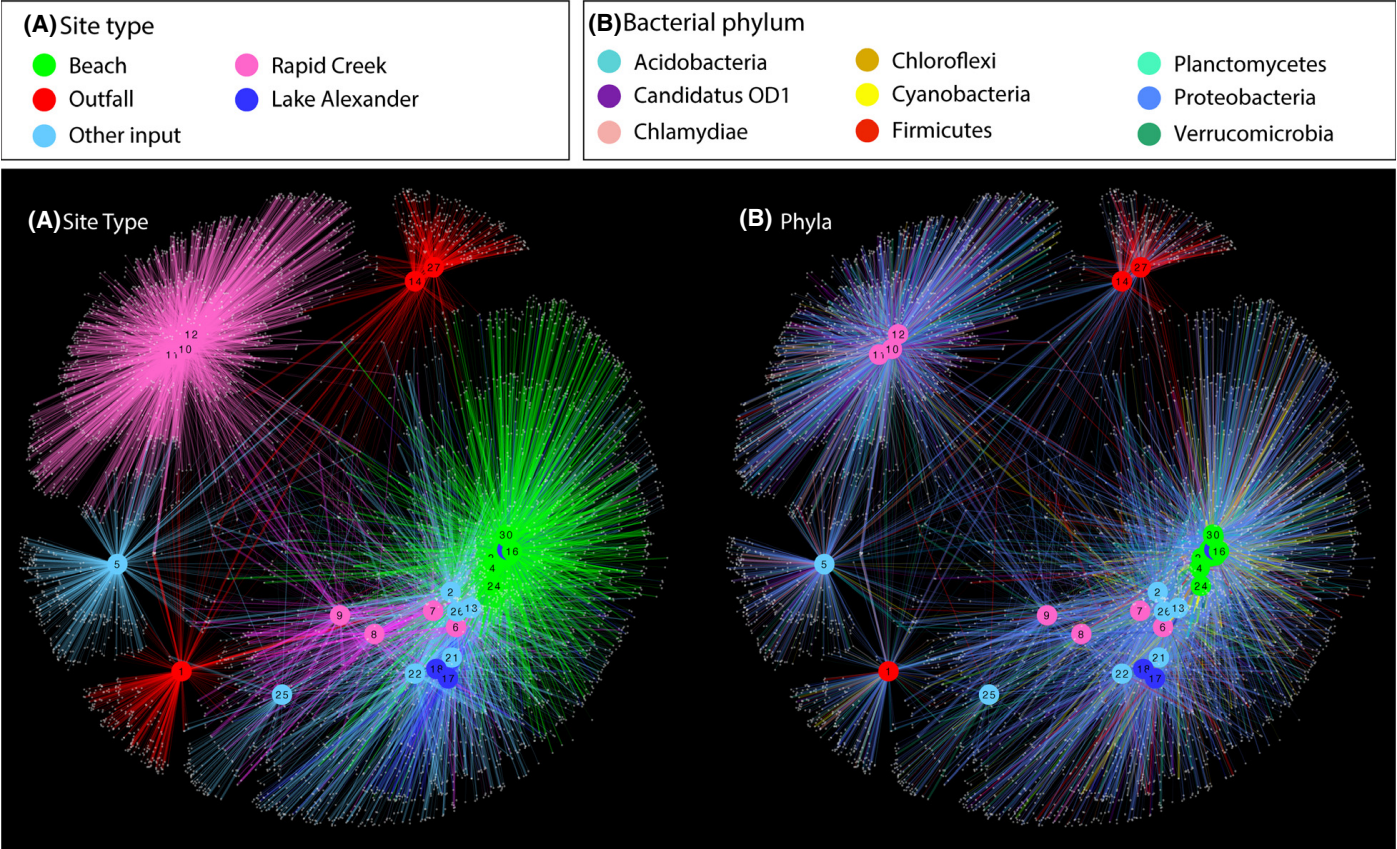
Network analysis of the top 5000 most abundant OTUs (97% similarity) in which the connecting edges are colored by site type (A) and bacterial phylum (B). Site nodes are consistently colored according to site type in both (A and B). The top 9 most abundant bacterial phyla were used in (B).

**Figure 7:**
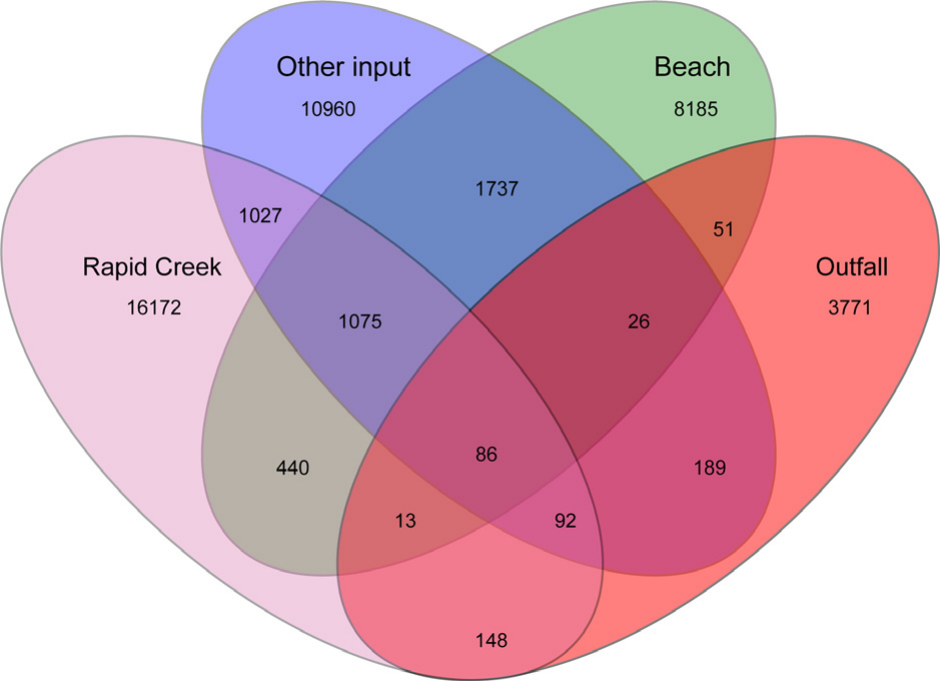
Venn diagram of shared OTUs (97% similarity) between site types. Lake Alexander was not drawn as it had a very similar OTU profile to the beaches. OTU, operational taxonomic units.

All of the beach sites had numerous OTUs in common, and they were also similar to many of the “other input” sites, Lake Alexander and lower Rapid Creek (Figs. 6A and 7). The upper reaches of Rapid Creek (sites 10, 11, and 12) were freshwater sites (Table S2) and their microbial communities were, not surprisingly, different (Fig. 6). Interestingly, two of the sewage outfalls had many OTUs in common with each other (sites 14 and 27) but the third site was different (site 1). This reflects the nutrient profiles of the outfalls (Table S3), in which nutrients were more similar at sites 14 and 27 compared to site 1. Although the differences were likely related to the various treatment processes used at the outfalls (see Experimental Procedures for description), the specific reason for this discrepancy is unclear. At the phylum level, most of the sites were dominated by members of the Proteobacteria, except for the two similar outfalls (sites 14 and 27), which contained many Firmicutes bacteria (Fig. 6B).

The estimated contribution of each of the suspected inputs to the beach bacterial communities was determined using the Bayesian source estimation program SourceTracker (Knights et al. 2011; Table 3). The outfall signature was detected at three beaches (sites 4, 20, and 29). In each case, the outfall contribution was estimated to be <1% of the community, which is not surprising, given the high microbial diversity of natural beach communities. The detection of the outfall signature at Lameroo Beach (site 29) further supports data from Figures 3 and 4 suggesting that the Larrakeyah outfall (site 27) influenced its surrounds. The outfall signature detected at site 4 (Rapid Ck Beach) was extremely low. Since Rapid Creek contained fecal OTUs (Fig. 3; Table 2) that were similar to the outfall OTUs, it is likely that these OTUs from Rapid Creek produced an ambiguity in the source classification. An outfall signature was also detected at site 20 (boat clubs), which had not been seen with previous tracking methods. This site is near a large number of moored boats and may be influenced by local sewage discharge, although further studies would be required to confirm this.

**Table 3:** Estimated proportion of each suspected source detected in the beach samples using 454 pyrosequencing

Beach no.	Location	Outfall	Other input	Lake Alexander	Rapid Creek	Unknown
3	Casuarina Beach	0	0.1899	0.6054	0.0165	0.1882
4	Rapid Ck Beach	0.0001	0.2999	0.3995	0.0934	0.2071
15	Fannie Bay bch	0	0.3260	0.4947	0.0001	0.1792
16	Fannie Bay bch	0	0.3974	0.3780	0	0.2246
20	Boat Clubs	0.0005	0.1463	0.7406	0	0.1126
23	Mindil Beach	0	0.1592	0.6687	0	0.1721
24	Little Mindil	0	0.2190	0.7106	0	0.0704
28	Doctors Gully	0	0.2484	0.6423	0	0.1093
29	Lameroo Beach	0.0013	0.1776	0.7164	0	0.1047
30	Wagait Beach	0	0.1968	0.6231	0	0.1801

The proportions were estimated using a Bayesian approach in SourceTracker (Knights et al. 2011).

The Rapid Creek signature was detected near the creek mouth (site 4) and could be detected ˜2 km to the north-east, at Casuarina Beach (site 3; Table 3). The other estimates from SourceTracker (Table 3) were less useful because SourceTracker is not bidirectional; that is, it cannot discriminate between environments that are both a source and a sink (Knights et al. 2011). For example, Lake Alexander was predicted as a large source for many of the beaches but this result is simply a reflection of the fact that Lake Alexander is a saltwater lake that naturally shares many OTUs with beaches, rather than being a source of OTUs.

**Figure 8:**
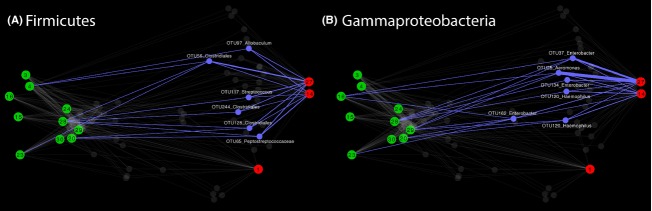
Network analysis of OTUs (97% similarity) shared between the outfalls and beaches for the Firmicutes (A) and Gammaproteobacteria (B). OTU node and edges have been highlighted in blue if they were detected in at least two outfalls and had an abundance of at least 100 in one of the sites. Beach sites are colored green and outfall sites are red. The classification of each OTU to the lowest confident level is written above the node. OTU, operational taxonomic units.

To more closely examine connections between sites (Figs. 6 and 7; Table 3), a network was drawn that contained only OTUs shared between outfalls and beaches (Fig. 8). Many of the most abundant shared OTUs are typically associated with sewage, such as Clostridiales, *Streptococcus*, *Peptostreptococcaceae*, *Aeromonas*, *Enterobacter*, and *Haemophilus* (Scott et al. 2005; McQuaig et al. 2012; McLellan et al. 2013; Newton et al. 2013; Shanks et al., 2013). Again, the Larrakeyah discharge (site 27) appeared to contribute bacteria to surrounding beaches (sites 28 and 29). Similar to the SourceTracker results, Rapid Creek Beach (site 4) contained potential fecal OTUs, and again several sites near Mindil Beach (sites 16, 20 and 23) were linked to fecal OTUs.

## Discussion

The Larrakeyah sewage outfall was a source of bacteria, including fecal bacteria, that impacted nearby beaches. This result was supported by many of the source-tracking approaches, including DGGE, specific marker genes and 454 pyrosequencing. Based on these data, this outfall is likely the source of high bacterial concentrations historically seen on Lameroo Beach. The outfall probably had this influence because the only treatment was by maceration, which is unlikely to remove many bacteria. In contrast, the two other sewage discharges, which employ enhanced primary and secondary treatments, had only minor impacts, and only on sites in very close proximity, suggesting that they are not a major source of bacteria.

Several sites along Mindil Beach had similar enterococci community profiles to those in adjacent drains and creeks, suggestive of a source-sink relationship. This result is of interest because the Mindil beaches have recorded high fecal indicator bacteria in the past, and the nearby creeks were a suggested source. On our sampling day, the adjacent creeks did indeed have high fecal bacteria counts and were frequently positive in the fecal PCR tests; however, results from the Mindil beaches were more complicated. Elevated fecal bacteria counts were detected on Mindil Beach, but other nearby beaches had low counts, and few of the Mindil beaches were positive in the fecal PCR tests. Using network analyses of 454 pyrosequencing data, several suspected fecal OTUs were sporadically detected on Mindil beaches but the pattern was not conclusive. It may be that the creeks only had a minor influence on the Mindil beaches, or contamination may not have occurred on our particular sampling day. Further studies are required to clarify this relationship.

An urban creek to Darwin's north-east (Rapid Creek) was a hotspot of fecal indicators and at two sites, likely human fecal pollution. The enterococci community profile was similar along the creek but different to other sites, indicating a local source. The nearby Rapid Creek Beach has periodically been closed in the past due to high fecal indicator counts, however, on our sampling day fecal bacteria counts at the beach were low, and enterococci profiles did not link the beach to the creek, suggesting that Rapid Creek was not discharging fecal bacteria. We did find, however, the Rapid Creek signature on Rapid Creek Beach using 454 pyrosequencing, indicating at least some bacterial transfer. Although additional sampling days are required to clarify this relationship.

Generally, connectivity between the sites was only seen within distinct geographical areas and it appears that most of the bacterial contamination on Darwin beaches is confined to local sources. In other catchments, the removal of localized contamination sources significantly improved water quality and reduced the frequency of beach closures (Dickerson et al. 2007; Korajkic et al. 2011).

We used DGGE on *E. coli* and enterococci communities, specific fecal markers and 454 pyrosequencing to track contamination sources. The DGGE signature for *E. coli* was complicated and variable, probably because *E. coli* strains occur in many different hosts and can survive outside the host and regrow in marine environments (Winfield and Groisman 2003; Layton et al. 2009; Whitman et al. 2009; Santiago-Rodríguez et al. 2010). While *E. coli* may continue to be useful in some source-tracking studies (Sigler and Pasutti 2006; Esseili et al. 2008), the complexity of the Darwin in-shore catchment was too great for these genes to be useful. On the other hand, an enterococci-targeted DGGE was developed and proved to be suitable for clarifying site connections. While many microbial source-tracking studies have examined enterococci concentration and specific enterococci genes (for review see Harwood et al. 2013) or examined enterococci community structure using pulsed-field gel electrophoresis (Kim et al. 2010; Furukawa et al. 2011) and amplified fragment length polymorphism (Burtscher et al. 2006), few studies have employed DGGE. We found that enterococci-targeted DGGE produced consistent site groupings (with some exceptions) that were reliable across replicates and complemented other source-tracking approaches. This reliability of the enterococci signal suggests that little variability or “noise” was introduced from environmental enterococci strains, potentially because environmental strains were not abundant in the tropical Darwin catchment. The DGGE technique has some limitations in that only abundant members of the community can be examined and it is often difficult to produce identical gel gradients, making it challenging to replicate results (Nocker et al. 2007). Nevertheless, with the appropriate selection of genes and conditions, this technique may be useful for future microbial source-tracking studies.

*Bacteroides thetaiotaomicron* was the most useful fecal maker in our study because it had high sensitivity to the three sewage outfalls, and required no intermediate culturing step. This marker has high specificity to human sewage and little cross-reactivity with other animals (Srinivasan et al. 2011; Aslan and Rose 2013) and our results suggest that it is useful for tropical catchments. The *Aeromonas* spp. marker was valuable because it is not only a fecal marker but also a pathogen marker (Singh et al. 2010). Sequence analysis of the positive results revealed the detection of the pathogen *A. hydrophilus* (Agger et al. 1985) and not an environmental strain. *A. hydrophilus* produces aerolysin which causes infections and septicemia (Singh et al. 2010). The inclusion of pathogen markers is an important component of microbial source tracking as it not only confirms the presence of sewage, but also the presence of human health risks. The final marker, *E. faecium*, is no longer considered human specific (Layton et al. 2009, 2010) and required an intermediate culturing step, reducing its usefulness.

Network analysis and source predictions using SourceTracker (Knights et al., 2011) of the 454 pyrosequencing data provided valuable information for understanding relationships between our sites. This approach was more sensitive than our other, more traditional source-tracking approaches and allowed us to detect lower levels of contamination. For example, Rapid Creek Beach was not linked to Rapid Creek using traditional approaches, despite their close proximity. However, SourceTracker predicted that almost 10% of OTUs on Rapid Creek Beach originated, in fact, from Rapid Creek, and network analysis allowed us to detect several suspected fecal OTUs on Rapid Creek Beach. Another example is the Mindil Beach sites, in which several suspected faecal OTUs were predicted using SourceTracker and identified by network analysis. These examples highlight the usefulness of high throughput sequencing approaches, which are likely to be used more prevalently for microbial source tracking as they decrease in cost and become more available. As was found in this study, high throughput sequencing approaches are especially useful for the development of markers specific to a particular system (Unno et al. 2010; Jeong et al. 2011).

## Conclusions

Practical and accurate microbial source-tracking techniques are extremely valuable for resource managers, particularly in rapidly expanding tropical population centers. Here, we show that enterococci community structure, fecal-specific markers and 454 pyrosequencing can be combined to identify potential sources of contamination in a tropical harbour. These multiple lines of evidence were an important part of discovering potential fecal markers in Darwin Harbour, and these results can now be used to develop more rapid monitoring techniques in order to reduce costs and turnaround time. One Darwin sewage outfall was a likely source of bacteria for nearby beaches, however, two other sewage outfalls had little impact. Several urban creeks and drains were also identified as potential contributors of bacteria. Connections between sites were generally confined to distinct locations, suggesting that contaminating bacteria were mostly derived from local sources. In this study, samples were collected at one dry-season sampling time. Bacterial communities are very likely to change during the wet season when increased rainfall reduces salinity, sediment is disturbed, groundwater is released and stormwater drains are active (McLellan et al. 2010; Passerat et al. 2011; Sidhu et al. 2013). It is recommended that future experiments measure changes throughout the year, especially during the wet-season.

## Supporting Information

**Table S1.** PCR information for genes used in the direct PCR tests and for denaturing gradient gel electrophoresis (DGGE). **Table S2.** Water quality data for each of the study sites. **Table S3.** Nutrient concentrations at each of the study sites. **Figure S1.** DGGE separation of the *mdh* gene in *E. coli* enriched water samples. Duplicate samples are only shown if they are different. Beaches are green, Lake Alexander is dark blue, other inputs are light blue, Rapid Ck is pink and the discharges are red. The branch numbers signify the cophenetic correlation value. **Figure S2.** DGGE separation of the *uid-A* gene in *E. coli* enriched water samples. Duplicate samples are only shown if they are different. Beaches are green, Lake Alexander is dark blue, other inputs are light blue, Rapid Ck is pink and the discharges are red. The branch numbers signify the cophenetic correlation value. **Figure S3.** DGGE separation of the *phoE* gene in *E. coli* enriched water samples. Duplicate samples are only shown if they are different. Beaches are green, Lake Alexander is dark blue, other inputs are light blue, Rapid Ck is pink and the discharges are red. The branch numbers signify the cophenetic correlation value. **Figure S4.** Rarefaction curves for number of operational taxonomic units (OTUs) in the 454 pyrosequencing dataset, for each of the sample categories.

## References

[b1] Agger WA, McCormick JD, Gurwith MJ (1985). Clinical and microbiological features of *Aeromonas hydrophila*-associated diarrhea. J. Clin. Microbiol.

[b2] AHU, Aquatic Health Unit (2012).

[b3] APHA, American Public Health Association (1989).

[b54] Arumugam M, Raes J, Pelletier E, Le Paslier D, Yamada T, Mende DR (2011). Enterotypes of the human gut microbiome. Nature.

[b4] Aslan A, Rose JB (2013). Evaluation of the host specificity of Bacteroides thetaiotaomicron alpha-1-6, mannanase gene as a sewage marker. Lett. Appl. Microbiol.

[b5] Burtscher MM, Köllner KE, Sommer R, Keiblinger K, Farnleitner AH, Mach RL (2006). Development of a novel amplified fragment length polymorphism (AFLP) typing method for enterococci isolates from cattle faeces and evaluation of the single versus pooled faecal sampling approach. J. Microbiol. Methods.

[b6] Claesson MJ, Wang Q, O'Sullivan O, Greene-Diniz R, Cole JR, Ross RP (2010). Comparison of two next-generation sequencing technologies for resolving highly complex microbiota composition using tandem variable 16S rRNA gene regions. Nucleic Acids Res.

[b7] Dickerson JW, Hagedorn C, Hassall A (2007). Detection and remediation of human-origin pollution at two public beaches in Virginia using multiple source tracking methods. Water Res.

[b8] Dobrindt U, Hochhut B, Hentschel U, Hacker J (2004). Genomic islands in pathogenic and environmental microorganisms. Nat. Rev. Microbiol.

[b9] Edgar RC, Haas BJ, Clemente JC, Quince C, Knight R (2011). UCHIME improves sensitivity and speed of chimera detection. Bioinformatics.

[b10] Esseili MA, Kassem II, Sigler V (2008). Optimization of DGGE community fingerprinting for characterizing *Escherichia coli* communities associated with fecal pollution. Water Res.

[b11] Furukawa T, Takahashi H, Yoshida T, Suzuki Y (2011). Genotypic analysis of Enterococci isolated from fecal-polluted water from different sources by pulsed-field gel electrophoresis (PFGE) for application to microbial source tracking. Microbes Environ.

[b12] Geoscience Australia (2006).

[b13] Harwood VJ, Staley C, Badgley BD, Borges K, Korajkic A (2013). Microbial source tracking markers for detection of fecal contamination in environmental waters: relationships between pathogens and human health outcomes. FEMS Microbiol. Rev.

[b14] Janda JM (1991). Recent advances in the study of the taxonomy, pathogenicity, and infectious syndromes associated with the genus *Aeromonas*. Clin. Microbiol. Rev.

[b15] Janda JM, Abbott SL (1998). Evolving concepts regarding the genus *Aeromonas*: an expanding panorama of species, disease presentations, and unanswered questions. Clin. Infect. Dis.

[b16] Jeong J-Y, Park H-D, Lee K-H, Weon H-Y, Ka J-O (2011). Microbial community analysis and identification of alternative host-specific fecal indicators in fecal and river water samples using pyrosequencing. J Microbiol.

[b17] Kassem II, Esseili MA, Sigler V (2011). Detection and differentiation of staphylococcal contamination of clinical surfaces using denaturing gradient gel electrophoresis. J. Hosp. Infect.

[b18] Ke D, Picard FJ, Martineau F, Ménard C, Roy PD, Ouellette M (1999). Development of a PCR assay for rapid detection of enterococci. J. Clin. Microbiol.

[b19] Kim S-Y, Lee JE, Lee S, Lee HT, Hur H-G, Ko G (2010). Characterization of Enterococcus spp. from human and animal feces using 16S rRNA sequences, the esp gene, and PFGE for microbial source tracking in Korea. Environ. Sci. Technol.

[b20] Knights D, Kuczynski J, Charlson ES, Zaneveld J, Mozer MC, Collman RG (2011). Bayesian community-wide culture-independent microbial source tracking. Nat. Methods.

[b21] Korajkic A, Brownell MJ, Harwood VJ (2011). Investigation of human sewage pollution and pathogen analysis at Florida Gulf coast Beaches. J. Appl. Microbiol.

[b22] Layton BA, Walters SP, Boehm AB (2009). Distribution and diversity of the enterococcal surface protein (*esp*) gene in animal hosts and the Pacific coast environment. J. Appl. Microbiol.

[b23] Layton BA, Walters SP, Lam LH, Boehm AB (2010). Enterococcus species distribution among human and animal hosts using multiplex PCR. J. Appl. Microbiol.

[b24] McLellan SL, Huse SM, Mueller-Spitz SR, Andreishcheva EN, Sogin ML (2010). Diversity and population structure of sewage-derived microorganisms in wastewater treatment plant influent. Environ. Microbiol.

[b25] McLellan SL, Newton RJ, Vandewalle JL, Shanks OC, Huse SM, Eren AM (2013). Sewage reflects the distribution of human faecal *Lachnospiraceae*. Environ. Microbiol.

[b26] McQuaig S, Griffith J, Harwood VJ (2012). Association of fecal indicator bacteria with human viruses and microbial source tracking markers at coastal beaches impacted by nonpoint source pollution. Appl. Environ. Microbiol.

[b27] Mieszkin S, Furet JP, Corthier G, Gourmelon M (2009). Estimation of pig fecal contamination in a river catchment by real-time PCR using two pig-specific Bacteroidales 16S rRNA genetic markers. Appl. Environ. Microbiol.

[b28] Newton RJ, Vandewalle JL, Borchardt MA, Gorelick MH, McLellan SL (2011). Lachnospiraceae and Bacteroidales alternative fecal indicators reveal chronic human sewage contamination in an urban harbor. Appl. Environ. Microbiol.

[b29] Newton RJ, Bootsma MJ, Morrison HG, Sogin ML, McLellan SL (2013). A microbial signature approach to identify fecal pollution in the waters off an urbanized coast of Lake Michigan. Microb. Ecol.

[b30] Nocker A, Burr M, Camper A (2007). Genotypic microbial community profiling: a critical technical review. Microb. Ecol.

[b31] Parameswaran P, Jalili R, Tao L, Shokralla S, Gharizadeh B, Ronaghi M (2007). A pyrosequencing-tailored nucleotide barcode design unveils opportunities for large-scale sample multiplexing. Nucleic Acids Res.

[b32] Passerat J, Ouattara NK, Mouchel J-M, Vincent R, Servais P (2011). Impact of an intense combined sewer overflow event on the microbiological water quality of the Seine River. Water Res.

[b33] Quast C, Pruesse E, Yilmaz P, Gerken J, Schweer T, Yarza P (2013). The SILVA ribosomal RNA gene database project: improved data processing and web-based tools. Nucleic Acids Res.

[b58] Quince C, Lanzen A, Davenport RJ, Turnbaugh PJ (2011). Removing noise from pyrosequenced amplicons. BMC Bioinformatics.

[b34] R Core Team (2013). R: a language and environment for statistical computing.

[b35] Rana SW, Kumar A, Walia SK, Berven K, Cumper K, Walia SK (2011). Isolation of Tn*1546*-like elements in vancomycin-resistant *Enterococcus faecium* isolated from wood frogs: an emerging risk for zoonotic bacterial infections to humans. J. Appl. Microbiol.

[b36] Santiago-Rodríguez TM, Dávila C, González J, Bonilla N, Marcos P, Urdaneta M (2010). Characterization of Enterococcus faecalis-infecting phages (enterophages) as markers of human fecal pollution in recreational waters. Water Res.

[b37] Schloss PD, Westcott SL, Ryabin T, Hall JR, Hartmann M, Hollister EB (2009). Introducing mothur: open-source, platform-independent, community-supported software for describing and comparing microbial communities. Appl. Environ. Microbiol.

[b38] Scott TM, Rose JB, Jenkins TM, Farrah SR, Lukasik J (2002). Microbial source tracking: current methodology and future directions. Appl. Environ. Microbiol.

[b39] Scott TM, Jenkins TM, Lukasik J, Rose JB (2005). Potential use of a host associated molecular marker in *Enterococcus faecium* as an index of human fecal pollution. Environ. Sci. Technol.

[b55] Shanks OC, Newton RJ, Kelty CA, Huse SM, Sogin ML, McLellen SL (2013). Comparison of the microbial community structures of untreated wastewaters from different geographic locales. Appl. Environ. Microbiol.

[b40] Sidhu JPS, Ahmed W, Gernjak W, Aryal R, McCarthy D, Palmer A (2013). Sewage pollution in urban stormwater runoff as evident from the widespread presence of multiple microbial and chemical source tracking markers. Sci. Total Environ.

[b41] Sigler V, Pasutti L (2006). Evaluation of denaturing gradient gel electrophoresis to differentiate *Escherichia coli* populations in secondary environments. Environ. Microbiol.

[b42] Silkie SS, Nelson KL (2009). Concentrations of host-specific and generic fecal markers measured by quantitative PCR in raw sewage and fresh animal feces. Water Res.

[b43] Singh V, Somvanshi P, Rathore G, Kapoor D, Mishra BN (2010). Gene cloning, expression, and characterization of recombinant aerolysin from *Aeromonas hydrophila*. Appl. Biochem. Biotechnol.

[b44] Smoot M, Ono K, Ruscheinski J, Wang P-L, Ideker T (2011). Cytoscape 2.8: new features for data integration and network visualization. Bioinformatics.

[b45] Sogin ML, Morrison HG, Huber JA, Welch DM, Huse SM, Neal PR (2006). Microbial diversity in the deep sea and the underexplored “rare biosphere”. Proc. Natl. Acad. Sci. USA.

[b46] Soller JA, Schoen ME, Bartrand T, Ravenscroft JE, Ashbolt NJ (2010). Estimated human health risks from exposure to recreational waters impacted by human and non-human sources of faecal contamination. Water Res.

[b47] Srinivasan S, Aslan A, Xagoraraki I, Alocilja E, Rose JB (2011). Escherichia coli, enterococci, and Bacteroides thetaiotaomicron qPCR signals through wastewater and septage treatment. Water Res.

[b48] Teng L-J, Hsueh P-R, Huang Y-H, Tsai J-C (2004). Identification of *Bacteroides thetaiotaomicron* on the basis of an unexpected specific amplicon of universal 16S ribosomal DNA PCR. J. Clin. Microbiol.

[b49] Toledo-Hernandez C, Ryu H, Gonzalez-Nieves J, Huertas E, Toranzos GA, Santo Domingo JW (2013). Tracking the primary sources of fecal pollution in a tropical watershed in a one-year study. Appl. Environ. Microbiol.

[b50] Unno T, Jang J, Han D, Kim JH, Sadowsky MJ, Kim O-S (2010). Use of barcoded pyrosequencing and shared OTUs to determine sources of fecal bacteria in watersheds. Environ. Sci. Technol.

[b51] Whitman RL, Przybyla-Kelly K, Shively DA, Nevers MB, Byappanahalli MN (2009). Hand-mouth transfer and potential for exposure to *E. coli* and F+ coliphage in beach sand, Chicago, Illinois. J. Water Health.

[b52] Wickham H (2009). ggplot2: elegant graphics for data analysis.

[b53] Winfield MD, Groisman EA (2003). Role of nonhost environments in the lifestyles of *Salmonella* and *Escherichia coli*. Appl. Environ. Microbiol.

