# Supplementary material for: Multiple approaches to microbial source tracking in tropical northern Australia
Source: Microbiologyopen. 2014 Sep 16;3(6):860–74. doi: 10.1002/mbo3.209 (PMC4263510; doi:10.1002/mbo3.209)
Supplement: Supplementary file 1 — Table S1. PCR information for genes used in the direct PCR tests and for denaturing gradient gel electrophoresis (DGGE). Table S2. Water quality data for each of the study sites. Table S3. Nutrient concentrations at each of the study sites. Figure S1. DGGE separation of the mdh gene in E. coli enriched water samples. Duplicate samples are only shown if they are different. Beaches are green, Lake Alexander is dark blue, other inputs are light blue, Rapid Ck is pink and the discharges are red. The branch numbers signify the cophenetic correlation value. Figure S2. DGGE separation of the uid-A gene in E. coli enriched water samples. Duplicate samples are only shown if they are different. Beaches are green, Lake Alexander is dark blue, other inputs are light blue, Rapid Ck is pink and the discharges are red. The branch numbers signify the cophenetic correlation value. Figure S3. DGGE separation of the phoE gene in E. coli enriched water samples. Duplicate samples are only shown if they are different. Beaches are green, Lake Alexander is dark blue, other inputs are light blue, Rapid Ck is pink and the discharges are red. The branch numbers signify the cophenetic correlation value. Figure S4. Rarefaction curves for number of operational taxonomic units (OTUs) in the 454 pyrosequencing dataset, for each of the sample categories. [file mbo30003-0860-sd1.docx]

**Supplementary Materials**

**Multiple Approaches to Microbial Source Tracking in Tropical Northern Australia**

Matthew Neave^1,2,*^, Heidi Luter^1,3^, Anna Padovan^1^, Simon Townsend^4^, Xavier Schobben^5^ and Karen Gibb^1^

^1^ Research Institute for the Environment and Livelihoods, Charles Darwin University, Casuarina, NT, Australia

^2^ Current address (Joint position): Woods Hole Oceanographic Institution, MA, USA and Red Sea Research Centre, KAUST, Saudi Arabia

^3^ Northern Australian Marine Research Alliance, Arafura Timor Research Facility Darwin, Brinkin, NT, Australia

^4^ Department of Land Resource Management, Northern Territory Government, Palmerston, NT, Australia.

^5^ Department of Health, Northern Territory Government, Casuarina NT, Australia.

* Corresponding author. Tel.: +1 508 524 5209; Email: [matthewjneave1@gmail.com](mailto:matthewjneave1@gmail.com), [mneave@whoi.edu](mailto:mneave@whoi.edu). Address: 266 Woods Hole Rd, Woods Hole, MA 02543, USA

| **Gene** | **Primer** | **Sequence** | **bp** | **Conditions** |
| --- | --- | --- | --- | --- |
| *Mdh*  (Hsu and Tsen, 2001) | mdh-f  mdh-r | 5’-ACTGAAAGGCAAACAGCCAAG-3’  5’-CGTTCTGTTCAAATGGCCTCAGG-3 | 392 | **First cycle**  94°C for 3 min  **35 cycles:**  94°C for 20 sec  60°C for 30 sec  72°C for 30 sec  **Final cycle:**  72°C for 5 min |
| *uidA-4*  (Bej *et al.*, 1991) | uidA-4-f  uidA-4-r | 5’-TATGGAATTTCGCCGATTTT-3’  5’-TGTTTGCCTCCCTGCTGCGG-3’ | 166 | **First cycle:**  95°C for 3 min  **25 cycles:**  94°C for 1 min  52°C for 2 min  72°C for 2 min  **Final cycle:**  72°C for 5 min |
| *phoE*  (Spierings *et al.*, 1993) | phoE-f  phoE-r | 5’-AAAGCCGTGGCACAGGCAAGCGT-3’  5’-TCAATTTGTTATCGCTATCCAGTTGG-3’ | 348 | **First cycle:**  95°C for 3 min  **35 cycles:**  95°C for 20 sec  55°C for 30 sec  72°C for 30 sec  **Final cycle:**  72°C for 5 min |
| *Tuf*  (Ke *et al.*, 1999) | Ent1  Ent2 | 5’-TACTGACAAACCATTCATGATG-3’  5’-AACTTCGTCACCAACGCGAAC-3’ | 112 | **First cycle:**  95°C for 3 min  **35 cycles:**  95°C for 20 sec  55°C for 30 sec  72°C for 30 sec  **Final cycle:**  72°C for 5 min |
| *Aerolysin* (*Aeromonas* spp.)  (Kong *et al.*, 2002) | Aero-f  Aero-r | 5’-TGTCGGSGATGACATGGAYGTG-3’  5’-CCAGTTCCAGTCCCACCACTTCA-3’ | 720 | **First cycle**  94°C for 2 min  **35 cycles:**  94°C for 1 min  59°C for 1 min  72°C for 2.5 min  **Final cycle:**  72°C for 5 min |
| *esp (Enterococcus faecium)*  (Scott *et al.*, 2005) | esp-f  esp-r | 5’-TATGAAAGCAACAGCACAAGTT-3’  5’-ACGTCGAAAGTTCGATTTCC-3’ | 680 | **First cycle:**  94°C for 5 min  **35 cycles:**  94°C for 1 min  58°C for 1 min  72°C for 1 min  **Final cycle:**  72°C for 5 min |
| *16S rRNA* (*Bacteroides thetaiotaomicron*)  (Teng *et al.*, 2004) | B.theta-f  B.theta-r | 5’-AACAGGTGGAAGCTGCGGA-3’  5’-AGCCTCCAACCGCATCAA-3’ | 542 | **First cycle:**  94°C for 3 min  **35 cycles:**  94°C for 1 min  60°C for 30 sec  72°C for 1 min  **Final cycle:**  72°C for 6 min |

**Table S1.** PCR information for genes used in the direct PCR tests and for denaturing gradient gel electrophoresis (DGGE).

| **Site** | **Location** |  | **pH** | **EC (mS/cm)** | **Turbidity (NTU)** | **Temp (_°_C)** | **DO (mg/L)** | **DO (%)** | **Total suspended solids (mg/L)** |
| --- | --- | --- | --- | --- | --- | --- | --- | --- | --- |
| **1** | **Leanyer Sanderson outfall** |  | **7.44** | **0.542** | **170** | **21.48** | **6.36** | **71.8** | **81.3** |
| 2 | Buffalo Ck mouth |  | 7.66 | 50.5 | 46.9 | 20.68 | 6.62 | 90.3 | 43.4 |
| 3 | Casuarina Beach |  | 8.02 | 50.2 | 9.58 | 22.67 | 6.67 | 93.2 | 211 |
| 4 | Rapid Ck Beach carpark |  | 8.07 | 49.71 | 32.1 | 22.64 | 7.26 | 101 | 69.2 |
| 5 | Stormwater drain to Rapid Ck |  | 8.84 | 0.3471 | 24 | 25.99 | 9.49 | 100 | 4.89 |
| 6 | Rapid Ck 6 Mouth |  | 8.09 | 50.02 | 24.9 | 20.8 | 6.98 | 94.6 | 6.08 |
| 7 | CDU channel into Rapid Creek |  | 8.06 | 50.19 | 27.1 | 20.87 | 7.02 | 95.2 | 3.71 |
| 8 | Rapid Ck 4 |  | 7.72 | 41.61 | 25 | 20.78 | 6.03 | 78.7 | 8.76 |
| 9 | Rapid Ck 5 ds Tide Gauge |  | 7.61 | 39.76 | 32.3 | 20.99 | 5.78 | 75.3 | 4.43 |
| 10 | Rapid Ck 5 ds McMillans Road |  | 5.68 | 0.0434 | 21.1 | 21.17 | 7.61 | 85.5 | 1.53 |
| 11 | Rapid Ck 1 near Airport |  | 5.42 | 0.0359 | 20.2 | 20.37 | 7.41 | 82.2 | 2.02 |
| 12 | Rapid Ck 4 Yankee Pool |  | 5.33 | 0.0364 | 22.3 | 19.39 | 6.47 | 70.6 | 37.5 |
| 13 | Ludmilla Ck mouth |  | 7.95 | 51 | 50.5 | 21.52 | 6.26 | 86.8 | 12.5 |
| **14** | **Ludmilla outfall** |  | **7.79** | **0.863** | **185** | **28.47** | **3.54** | **45.4** | **111** |
| 15 | Fannie Bay bch |  | 8.13 | 51.54 | 4 | 23.25 | 7.68 | 110.3 | 10.5 |
| 16 | Fannie Bay bch |  | 8.11 | 51.6 | 6 | 23.32 | 7.58 | 109.2 | 15.2 |
| 17 | Lake Alexander NE |  | 8.49 | 48.84 | 3 | 23.36 | 9.74 | 136.3 | 5.92 |
| 18 | Lake Alexander SW |  | 8.4 | 49.16 | 2.8 | 23.21 | 8.74 | 123.8 | 7.95 |
| 19 | Lake Alexander intake |  | 8.12 | 51.45 | 3.4 | 23.19 | 7.69 | 160.2 | 9.16 |
| 20 | Boat Clubs |  | 8.64 | 51.8 | 3.89 | 22.79 | 7.27 | 104.2 | 4.56 |
| 21 | Vesteys Ck |  | 8.03 | 47.98 | 1.2 | 22.68 | 7.07 | 99.4 | 2.88 |
| 22 | Botanic Garden Drain |  | 7.78 | 45.84 | 2.2 | 19.98 | 5.94 | 80 | 4.05 |
| 23 | Mindil Beach |  | 8.07 | 50.01 | 10 | 21.8 | 7.49 | 104.9 | 24.2 |
| 24 | Little Mindil |  | 8.05 | 49.18 | 3.2 | 21.12 | 7.36 | 101.5 | 3.69 |
| 25 | Golf Pond |  | 8.81 | 17.14 | 1.82 | 21.81 | 10.55 | 127.9 | 9.33 |
| 26 | Cullen Bay |  | 8 | 49.8 | 3.4 | 22.34 | 7.24 | 101.8 | 8.10 |
| **27** | **Larrakeyah discharge** |  | **7.66** | **1.91** | **265** | **28.24** | **2.93** | **37.01** | **128** |
| 28 | Doctors Gully |  | 8.25 | 51.6 | 4.4 | 22.18 | 6.73 | 95.4 | 7.04 |
| 29 | Lameroo Beach |  | 8.2 | 51.6 | 4.67 | 22.35 | 6.78 | 96.9 | 5.05 |
| 30 | Wagait Beach |  | 8.61 | 51.6 | 10.6 | 22.28 | 8.12 | 105.6 | 10.3 |

**Table S2.** Water quality data for each of the study sites

**Table S3.** Nutrient concentrations at each of the study sites

| **Site** | **Location** | **Nitrite (ppb)** | **Nitrate (ppb)** | **Ammonia (ppb)** | **Total nitrogen (ppb)** | **Total phosphorous (ppb)** |
| --- | --- | --- | --- | --- | --- | --- |
| **1** | **Leanyer Sanderson outfall** | **1770.00** | **570.46** | **4132.80** | **10407.5** | **839.72** |
| 2 | Buffalo Ck mouth | 2.44 | 2.78 | 14.42 | 127.5 | 31.41 |
| 3 | Casuarina Beach | 1.50 | 3.40 | 14.82 | 107.5 | 25.83 |
| 4 | Rapid Ck Beach carpark | 2.09 | 1.00 | 10.17 | 97.5 | 26.72 |
| 5 | Stormwater drain to Rapid Ck | 1.19 | 81.68 | 4.00 | 207.5 | 282.64 |
| 6 | Rapid Ck 6 Mouth | 2.02 | 2.08 | 5.23 | 97.5 | 25.63 |
| 7 | CDU channel into Rapid Creek | 2.19 | 20.38 | 5.06 | 117.5 | 23.47 |
| 8 | Rapid Ck 4 | 1.60 | 20.89 | 8.01 | 137.5 | 20.32 |
| 9 | Rapid Ck 5 ds Tide Gauge | 1.63 | 23.15 | 12.16 | 127.5 | 13.21 |
| 10 | Rapid Ck 5 ds McMillans Road | 0.50 | 7.09 | 8.70 | 37.5 | 1.50 |
| 11 | Rapid Ck 1 near Airport | 0.50 | 15.21 | 9.67 | 77.5 | 1.50 |
| 12 | Rapid Ck 4 Yankee Pool | 0.50 | 2.32 | 9.08 | 27.5 | 1.50 |
| 13 | Ludmilla Ck mouth | 2.19 | 9.18 | 19.14 | 127.5 | 28.04 |
| **14** | **Ludmilla outfall** | **63.90** | **24.21** | **33481.8** | **42507.5** | **4916.69** |
| 15 | Fannie Bay bch | 2.52 | 8.28 | 16.99 | 217.5 | 26.94 |
| 16 | Fannie Bay bch | 2.10 | 8.89 | 17.34 | 107.5 | 25.25 |
| 17 | Lake Alexander NE | 2.42 | 1.00 | 13.10 | 197.5 | 22.21 |
| 18 | Lake Alexander SW | 2.24 | 2.62 | 5.18 | 207.5 | 23.77 |
| 19 | Lake Alexander intake | 2.01 | 4.09 | 7.40 | 107.5 | 24.67 |
| 20 | Boat Clubs | 1.92 | 2.60 | 19.71 | 97.5 | 25.46 |
| 21 | Vesteys Ck | 2.06 | 3.33 | 11.02 | 107.5 | 26.54 |
| 22 | Botanic Garden Drain | 2.85 | 29.89 | 13.76 | 227.5 | 28.38 |
| 23 | Mindil Beach | 2.39 | 1.00 | 11.04 | 87.5 | 25.27 |
| 24 | Little Mindil | 2.14 | 1.00 | 12.82 | 97.5 | 25.96 |
| 25 | Golf Pond | 0.50 | 4.54 | 1.50 | 497.5 | 13.73 |
| 26 | Cullen Bay | 2.12 | 1.00 | 8.95 | 137.5 | 29.02 |
| **27** | **Larrakeyah discharge** | **39.40** | **1.00** | **36416.2** | **46457.5** | **5799.00** |
| 28 | Doctors Gully | 2.22 | 2.34 | 18.66 | 107.5 | 26.93 |
| 29 | Lameroo Beach | 2.16 | 3.01 | 16.69 | 97.5 | 27.54 |
| 30 | Wagait Beach | 1.98 | 4.34 | 8.69 | 92.5 | 26.94 |


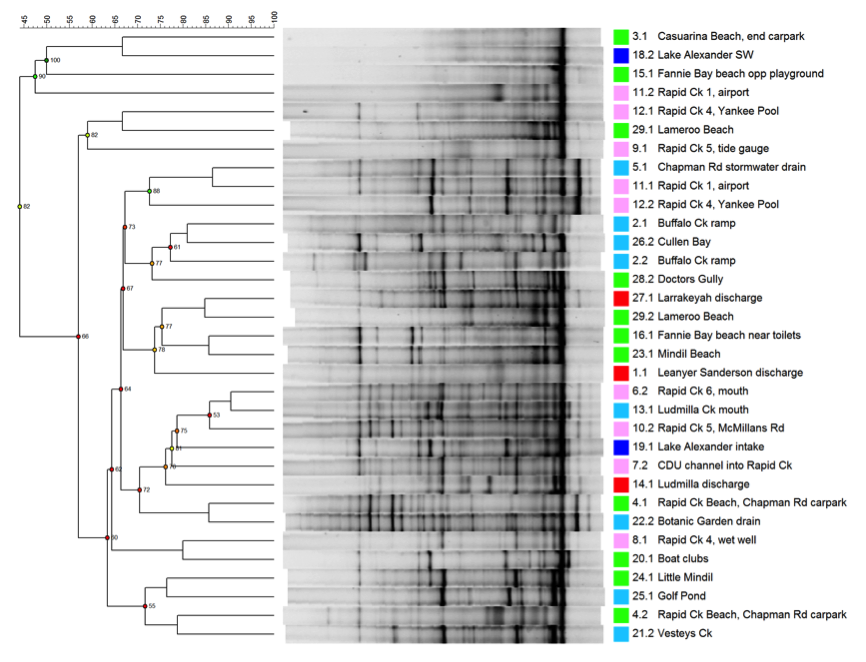


**Fig. S1.** DGGE separation of the *mdh* gene in *E. coli* enriched water samples. Duplicate samples are only shown if they are different. Beaches are green, Lake Alexander is dark blue, other inputs are light blue, Rapid Ck is pink and the discharges are red. The branch numbers signify the cophenetic correlation value.


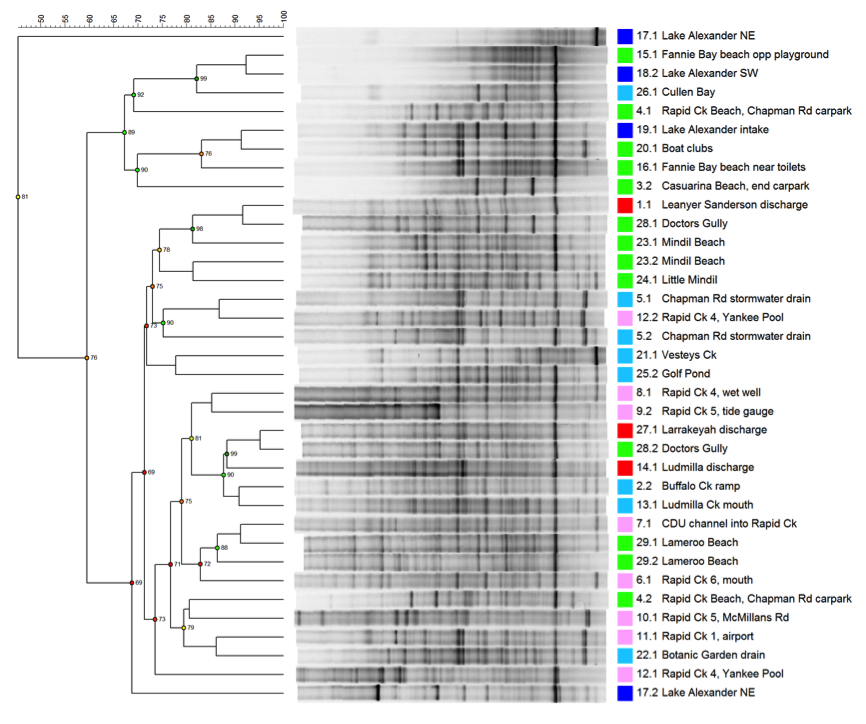
**Fig. S2.** DGGE separation of the *uid-A* gene in *E. coli* enriched water samples. Duplicate samples are only shown if they are different. Beaches are green, Lake Alexander is dark blue, other inputs are light blue, Rapid Ck is pink and the discharges are red. The branch numbers signify the cophenetic correlation value.


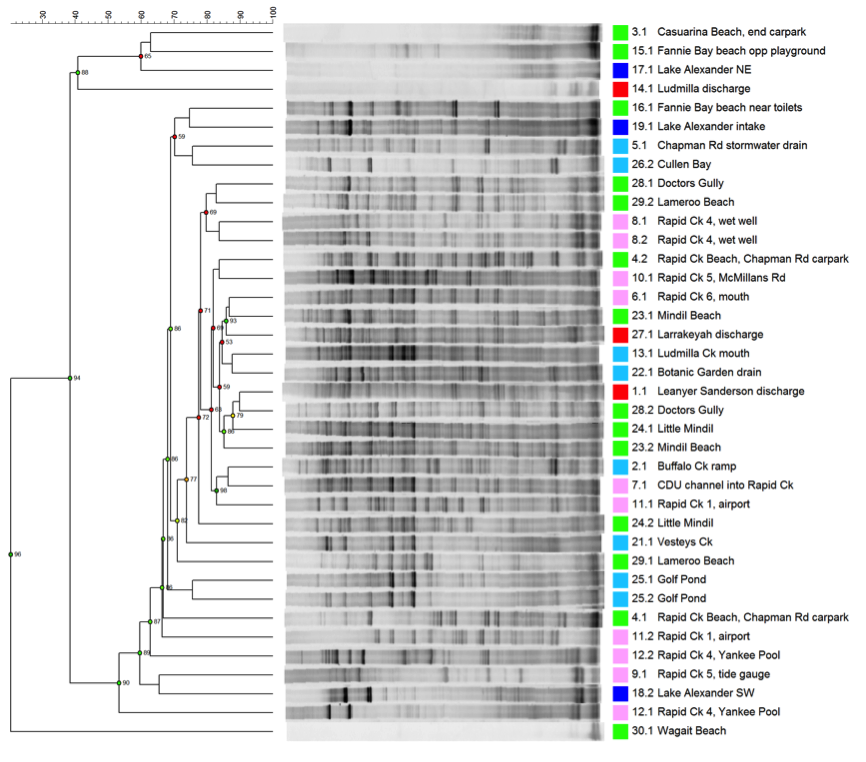


**Fig. S3.** DGGE separation of the *phoE* gene in *E. coli* enriched water samples. Duplicate samples are only shown if they are different. Beaches are green, Lake Alexander is dark blue, other inputs are light blue, Rapid Ck is pink and the discharges are red. The branch numbers signify the cophenetic correlation value.


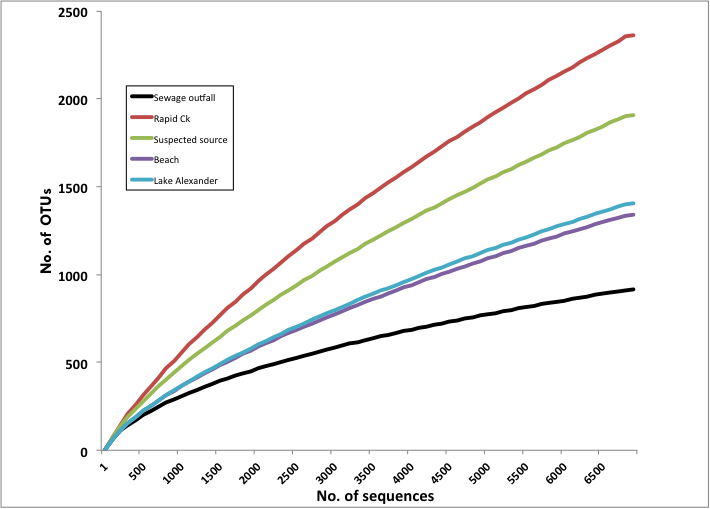


**Fig. S4.** Rarefaction curves for number of operational taxonomic units (OTUs) in the 454 pyrosequencing dataset, for each of the sample categories.

**References**

Bej, A.K., DiCesare, J.L., Haff, L., and Atlas, R.M. (1991) Detection of *Escherichia coli* and *Shigella* spp. in water by using the polymerase chain reaction and gene probes for uid. *Appl Environ Microbiol* **57**: 1013-1017.

Hsu, S.C., and Tsen, H.Y. (2001) PCR primers designed from malic acid dehydrogenase gene and their use for detection of *Escherichia coli* in water and milk samples. *Int J Food Microbiol* **64**: 1-11.

Ke, D., Picard, F.J., Martineau, F., Ménard, C., Roy, P.D., Ouellette, M., and Bergeron, M.G. (1999) Development of a PCR assay for rapid detection of enterococci. *J Clin Microbiol* **37**: 3497-3503.

Kong, R.Y.C., Lee, S.K.Y., Law, T.W.F., Law, S.H.W., and Law, R.S.S. (2002) Rapid detection of six types of bacterial pathogens in marine waters by multiplex PCR. *Water Res* **36**: 2802-2812.

Scott, T.M., Jenkins, T.M., Lukasik, J., and Rose, J.B. (2005) Potential use of a host associated molecular marker in *Enterococcus faecium* as an index of human fecal pollution. *Environ Sci Technol* **39**: 283-287.

Spierings, G.C., Ockhuijsen, C., Hofstra, H., and Tommassen, J. (1993) Polymerase chain reaction for the specific detection of *Escherichia coli*/*Shigella*. *Res Microbiol* **144**: 557-564.

Teng, L.-J., Hsueh, P.-R., Huang, Y.-H., and Tsai, J.-C. (2004) Identification of *Bacteroides thetaiotaomicron* on the basis of an unexpected specific amplicon of universal 16S ribosomal DNA PCR. *J Clin Microbiol* **42**: 1727-2730.
